# Current Concepts of Psoriasis Immunopathogenesis

**DOI:** 10.3390/ijms222111574

**Published:** 2021-10-26

**Authors:** Marijana Vičić, Marija Kaštelan, Ines Brajac, Vlatka Sotošek, Larisa Prpić Massari

**Affiliations:** 1 Department of Dermatovenereology, Medical Faculty University of Rijeka, Clinical Hospital Center Rijeka, Krešimirova 42, 51000 Rijeka, Croatia; marijana.vicic@medri.uniri.hr (M.V.); marijakastelan@yahoo.com (M.K.); ines.brajac@medri.uniri.hr (I.B.); 2 Department of Anesthesiology, Reanimation and Intensive Care, Medical Faculty University of Rijeka, Clinical Hospital Center Rijeka, Tome Strižića 3, 51000 Rijeka, Croatia; vlatkast@medri.uniri.hr

**Keywords:** psoriasis, etiology, immunopathogenesis, T lymphocytes, dendritic cells, keratinocytes, macrophages, NK cells, NKT cells, IL-23/Th17 pathway

## Abstract

Psoriasis is a recurrent, chronic, immune-mediated, systemic inflammatory disease of the skin, joints, and other organic systems. After atopic dermatitis, chronic stationary psoriasis is the most common inflammatory skin disease, affecting an average of 2–4% of the world’s population. The disease carries a significant burden due to its numerous comorbidities and the major impact on patients’ social and emotional aspects of life. According to current knowledge, psoriasis is a multifactorial disease that occurs in genetically predisposed individuals under various environmental factors, which trigger an immune response disorder with a series of complex inflammatory cascades. The disease is initiated and maintained by mutual interaction of the innate and adaptive immune cells, primarily dendritic cells, T lymphocytes, and keratinocytes, whose leading role alternates at different stages of the disease, consisting mainly in the IL-23/Th17 pathway. Inflammatory events result in consequent epidermal and dermal changes and evolution of the characteristic psoriatic phenotype, respectively. This paper aims to present a comprehensive overview of current knowledge on psoriasis genetic and environmental etiological factors, immunopathogenesis, and the leading cellular and cytokine participants in the inflammatory pathways of this disease.

## 1. Introduction

Psoriasis is a chronic, recurrent, immune-mediated, inflammatory skin disease that is characterized by the clinical appearance of sharply demarcated, erythematous papules or plaques covered with silvery-white scales [1]. Psoriasis is, after atopic dermatitis, the most common inflammatory skin disease, whose incidence has been slightly increasing in the last three decades [2]. The prevalence of psoriasis significantly varies among different populations and ranges from 0.24% in Taiwan to 8.5% in Norway, while the disease is unknown in South American Indians [3,4]. On average, psoriasis affects about 2–4% of the world’s population [2]. People of both sexes suffer equally, while the disease is present mainly in adults and most often occurs in two age groups, between 20 and 30 years and 50 and 60 years [2].

In the past, psoriasis was considered an exclusive skin disease, while today, it is commonly understood as a systemic inflammatory disease [1]. Systemic inflammation is caused by the action of the “psoriatic march”, representing the abundance of proinflammatory cytokines not only in lesional psoriatic skin but in patients’ circulation as well [5]. The same mechanism contributes to the development of concomitant diseases, where 73% of patients, especially those with severe psoriasis, have at least one comorbidity [6]. The most common of these are psoriatic arthritis and Crohn’s disease, which share pathogenetic mechanisms with psoriasis [7,8]. Still, there is also an increased risk of metabolic syndrome [9], nonalcoholic fatty liver disease [10], cardiovascular [11,12], respiratory [13,14,15], and autoimmune diseases, such as Hashimoto’s thyroiditis, autoimmune hepatitis, multiple sclerosis [5], malignancies, especially T-cell skin lymphoma [7], and mental disorders, most commonly depression and anxiety [7,16]. Severe disease has been shown to increase overall mortality and reduce life expectancy by 3.5 years in men and 4.4 years in women, compared to those without psoriasis [17,18]. The most common specific causes of death in psoriasis patients are cardiovascular incidents followed by infections, malignancies, liver, kidney, respiratory, and digestive system diseases [18,19].

According to today’s knowledge, psoriasis is a multifactorial disease caused by the interaction of genetic and environmental factors [20].

## 2. Genetic Factors in the Development of Psoriasis

In psoriasis, the polygenic model of inheritance predominates [21]. Studies of affected families have revealed chromosomal areas associated with the onset of the disease, called PSORS (from the psoriasis susceptibility locus) [22]. Although twelve such areas are known to date, the most significant is the PSORS1 region, which is responsible for 35–50% of inherited psoriasis cases [22]. It also contains the first gene associated with psoriasis, HLA-Cw6 (from human leukocyte antigen C), which has been found in 10.5–77.2% of patients [23] and which plays a key role in antigen presentation and regulation of cytotoxic T-cells’ (Tc) function [24]. Depending on HLA status, psoriasis is divided into two types [25]. Type I occurs in 65% of patients who, as carriers of HLA-Cw6, have a 9–23-fold higher risk of developing psoriasis with earlier onset and severe course, while their family history for the disease is positive [26]. Type II occurs in individuals older than 40 years, in whom the HLA-Cw6 allele is absent, and the clinical course of the disease is milder [23].

Most PSORS-region genes are unknown, wherefore genome-wide association studies (GWAS) have been conducted in recent years [22]. These studies examine the single nucleotide polymorphisms (SNPs) presence in cohorts of patients and controls, and they identified more than 50 additional regions at risk of disease [22]. While changes in one base slightly increase the risk of psoriasis, the combined action of several factors allows its development [27]. Significantly, only a small number of detected genes encode skin proteins, such as the epidermal differentiation complex (EDC), while most candidate genes encode proteins with roles in the innate and adaptive immune system, including tumor necrosis factor-alpha (TNF-α), nuclear factor kappa-light-chain-enhancer of activated B cells (NF-κB), interferon type I (IFN-I), interleukin (IL)-12 and 23, as well as those influencing the development and polarization of helper T17 lymphocytes (Th17) and cytotoxic T17 lymphocytes (Tc17) [21,22,28].

## 3. Environmental Factors in the Development of Psoriasis

Certain environmental stimuli, such as infections, can trigger the onset or worsen the existing psoriatic disease [24]. Streptococcal infection is associated with the development of guttate and chronic stationary psoriasis, while type I HIV contributes to the worsening of psoriasis [24]. Metabolic and hormonal changes can also influence the psoriasis course, for instance, hypocalcemia can cause pustular psoriasis and herpetiform impetigo, while hormonal status in pregnancy improves psoriasis in 40–50% of patients and worsens it in half of them in the postpartum period [29]. Drugs such as lithium, beta-blockers, angiotensin-converting enzyme (ACE) inhibitors, interferons, nonsteroidal antirheumatic drugs, and antimalarials may cause the onset or worsening of pre-existing disease, just as abrupt discontinuation of systemic corticosteroids may exacerbate plaque or pustular psoriasis [25]. Smoking and obesity contribute to the development of psoriasis, while the existing disease is exacerbated by alcohol consumption [25].

In about 25% of patients, mechanical trauma will cause the appearance of new psoriatic lesions on so far unaffected skin, which is known as the Köebner phenomenon [25]. It is more common in the active phase of the disease and severe forms of psoriasis and can be caused by injuries such as surgery, tattoos, injections, insect bites, burns, X-rays, and the use of irritants [25]. Although ultraviolet radiation commonly contributes to clinical improvement, in certain individuals, exposure to strong sunlight can stimulate the formation of so-called photosensitive psoriasis [25]. Mental stress is a well-known trigger, which has been confirmed to be able to initiate or worsen an existing illness [25]. These observations that skin conditions, such as psoriasis, can be induced or modified by the sum of environmental exposures during life are supported by the recently defined concept of skin exposome [30,31].

## 4. Immunopathogenesis of Psoriasis

**Figure 1:**
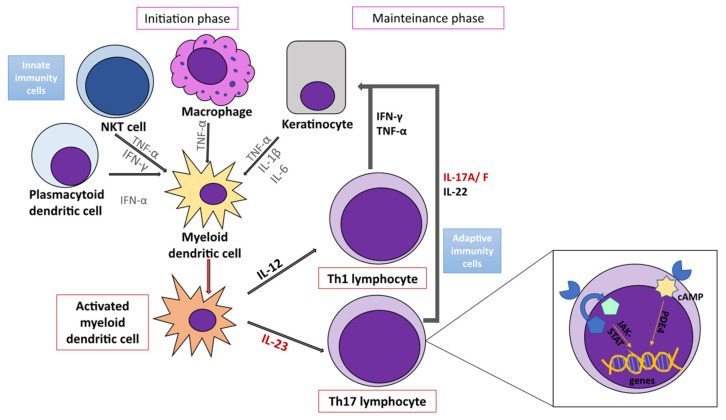
Major effector cells and signaling pathways in the immunopathogenesis of psoriasis. The immunopathogenesis of psoriasis involves a complex inflammatory cascade, which is initially triggered by innate immune cells (keratinocytes, dendritic cells, NKT cells, macrophages). At the same time, the disease progresses and is maintained by their interaction with adaptive immune cells (T lymphocytes). The central mechanism of the disease is the IL-23/Th17 axis, whose executive cytokines IL-22 and IL-17A/F lead to keratinocyte proliferation, production of proinflammatory cytokines, chemokines and AMP, and the formation of a positive feedback loop, which maintains the inflammatory process. Cytokines in cells activate signaling and transcription pathways (cAMP, JAK-STAT), which achieve increased transcription of messenger genes and cytokines involved in the disease pathogenesis. Adapted from: [36].

Traditionally, the development of psoriasis is interpreted by the initiation phase and the maintenance phase of the disease [3]. The proinflammatory cytokine cascade is triggered by plasmacytoid dendritic cell’s (pDC) stimulation by complexes of DNA and the antimicrobial peptide cathelicidin (LL-37), which is released by injured keratinocytes [3]. Likewise, damaged melanocytes can produce ADAMTS-like protein 5, the other possible autoantigen in psoriasis [32]. In response to the stimulus, pDCs secrete IFN-α, a key cytokine of the initiation phase [33]. It activates local myeloid dendritic cells (mDCs) and stimulates their migration to regional lymph nodes [33]. Other innate immune cells, i.e., keratinocytes, macrophages, and NKT cells, also contribute to mDCs activation by secreting INF-γ, TNF-α, IL-1-β, and IL-6 [34]. Activated mDCs then produce TNF-α, IL-12, and IL-23, which cause the differentiation and proliferation of naïve T lymphocytes into mature T1 (Th1 and Tc1), T17 (Th17 and Tc17), and T22 (Th22 and Tc22) lymphocytes, which enter the bloodstream and acquire the ability to populate the skin [27]. Activated T1 lymphocytes release IFN-γ and TNF-α, while T17 lymphocytes secrete the central executive proinflammatory cytokine IL-17A, which is additionally produced by γδ T lymphocytes, NK cells, mastocytes, and innate lymphoid cells (ILCs) [27]. The cytokines IL-22 and IL-17A/F, which are products of the IL-23/Th17 axis, cause proliferation and impaired differentiation of keratinocytes, developing a characteristic psoriatic phenotype [34]. At the same time, keratinocytes are not just passive observers but also respond to stimulation by secreting antimicrobial peptides (AMPs), cytokines, and chemokines, which promote further activation of T lymphocytes and mobilization of other inflammatory cells, primarily macrophages, dendritic cells, and neutrophils, thus stimulating the formation of chronic inflammation, i.e., phase of disease maintenance [3]. The inflammatory cascade promotes angiogenesis furthermore, which contributes to the additional migration of immune cells into the psoriatic lesion [33]. Ultimately, the cytokines’ effect is achieved by activating intracellular pathways, which act on the transcription of key messenger genes [35]. Thus INF-γ, IL-12, IL-22, and IL-23 activate the JAK-STAT (from Janus Kinases—Signal Transducer and Activator of Transcription proteins) pathway, whereas phosphodiesterase-4 (PDE-4) inhibits the anti-inflammatory action of the cAMP signaling molecule (from cyclic adenosine monophosphate) [36] (Figure 1).

### 4.1. Main Cells Involved in Psoriasis Inflammatory Networks

#### 4.1.1. Dendritic Cells

Dendritic cells (DCs) act as a link between innate and adaptive immunity and are undoubtedly one of the most important components in psoriasis development [34]. They contribute to the disease by performing the role of professional antigen-presenting cells (APCs), participating in the T lymphocytes’ activation and differentiation and by cytokine and chemokine production, thus enhancing the inflammatory process [34]. While epidermal Langerhans cells, together with dermal plasmacytoid and myeloid DCs, are present in healthy human skin, the latter two types contribute to the psoriasis pathogenesis [37].

Plasmacytoid dendritic cells (pDCs) trigger disease after a complex of keratinocyte DNA and antimicrobial peptide LL-37 binds to their toll-like-9 receptor (TLR-9) [38]. Afterwards, they respond by releasing large amounts of IFN type I, particularly IFN-α, which stimulate mDCs maturation and T lymphocytes’ activation, with a consequent inflammatory cascade that forms the psoriatic phenotype [34]. It has been confirmed that pDCs contribute to the disease pathogenesis as the main source of IFN-α in the skin, since the experimental blockade of this cytokine prevented the development of skin lesions [39]. While there are few pDCs in normal skin, their number is increased in lesional psoriatic skin [40].

Myeloid dendritic cells (mDCs) are recognizable by the presence of αx integrin CD11c, while two subpopulations are differentiated, depending on the blood dendritic cell antigen (BDCA) expression [34]. The first consists of BDCA-1-positive (CD1c+) or “resident” DCs, which, as mature APCs, perform the local presentation of antigen to T lymphocytes and whose number is equal in altered and unaltered psoriatic skin [34]. The second subpopulation consists of the most numerous CD11c+ cells in psoriatic skin, the so-called BDCA-1-negative (CD1c−) or “inflammatory” DC (iDC), whose number is thirtyfold increased in the lesional dermis and normalized by effective antipsoriatic therapy (e.g., etanercept, infliximab, UVB phototherapy) [38]. These cells, also known as TiP-DC (from TNF-α/iNOS producing), produce TNF-α and inducible nitric oxide synthase (iNOS), as well as IL-6, IL-12, IL-20, and IL-23, and play an essential role in maintaining and enhancing psoriatic inflammation, primarily by activating Th17 cells and managing the IL-17 response [38].

Although Langerhans cells (LCs) can present antigens in regional lymph nodes, their role in psoriasis has not yet been elucidated [34]. It is thought that they could be important in maintaining tolerance to antigens that appear in the skin. While the difference in the number of LCs has not been observed in the epidermis of lesional and nonlesional psoriatic and healthy skin, their reduced motility with consequent retention within the lesional epidermis contributes to the immune response disorder [40].

#### 4.1.2. Macrophages

Experimental mouse psoriasis models have confirmed that macrophages, as innate immune cells, contribute to the development and maintenance of psoriatic lesions [41]. Most mature skin macrophages have a pronounced CD163 marker [41]. Their number is three times larger in both sections of psoriatic skin and returns to normal levels after the use of effective antipsoriatic therapy [40]. Their role in psoriasis is not fully known; however, it is thought that they contribute to the pathogenesis by stimulating adaptive immunity, presenting antigens to T lymphocytes, and secreting inflammatory products, particularly TNF-α, IL-12, and IL-23 [41]. The macrophages’ flexibility and ability of their functional and phenotypic adaptation to the environment were confirmed after the discovery of new so-called M (IL-23) subpopulations, which respond to IL-23 stimuli by releasing IL-17A, IL-22, and IFN-γ [42,43]. Activated macrophages are also important in maintaining tissue homeostasis by phagocytosis and in regulating angiogenesis by releasing vascular endothelial growth factor (VEGF) [40].

#### 4.1.3. Lymphocytes

The development of psoriatic lesions is associated with different subpopulations of T lymphocytes that favor the disease pathogenesis by abnormal cellular activation, proinflammatory cytokines’ secretion, and immune cells’ mobilization [44]. Most lymphocytes in the skin are αβ memory CD45RO+ T lymphocytes, whereas helper (CD4+) and cytotoxic (CD8+) T lymphocytes form the two most numerous lymphocytes’ groups with the alpha-beta T-cell receptor (αβ-TCR) [45]. The number of T lymphocytes is increased in psoriatic skin biopsies, namely CD8+ in the epidermal and CD4+ in the perivascular areas of the upper dermal compartment [45]. An experiment with SCID mice, in which the psoriatic phenotype was developed after introducing CD4+ T lymphocytes into the transplant of unaffected patient’s skin, confirmed the necessity of dermal infiltration by these cells in triggering the pathogenic process [46]. However, the moment of CD8+ T lymphocytes’ epidermal entry is also crucial, since the disease will not develop if it is prevented or disabled by cell depletion [47]. Some CD4+ and CD8+ T lymphocytes, which are more numerous in the patients’ blood as well [34], will leave the circulation and enter the skin through the interaction of cutaneous lymphocyte antigen (CLA), expressed on psoriatic lymphocytes and E-selectin on skin capillaries [48]. Through α1β1 integrin or VLA-1 (from very late antigen-1), CD8+ lymphocytes bind to the basal membrane’s type IV collagen and enter the epidermis [49]. The mentioned mechanisms achieve significant infiltration of the lesional epidermis and dermis by CD4+ and CD8+ T lymphocytes [34].

Many pathogenic lymphocytes’ subpopulations have been detected by cytokines secreted in cellular infiltrates of affected psoriatic skin [34]. Thus, Th1 lymphocytes, whose differentiation is controlled by IL-12, are recognized by IFN-γ, TNF-α, and IL-2 cytokine production [34]. Th17 lymphocytes differentiate under the influence of IL-23, IL-1β, TGF-β and IL-6, secrete IL-17A, IL-17F, TNF-α, IL-21, IL-22, and IL-26, and they play a crucial role in the maintenance of psoriasis chronic inflammation [34]. TNF-α and IL-6 direct the differentiation of Th22, which produces IL-22, IL-13, and IL-26 [50]. Populations of cytotoxic T lymphocytes, which produce identical cytokines as their helper variants, are labeled as Tc1, Tc17, and Tc22 [34,51]. However, it is suggested that Tc lymphocytes exert their effector functions through the cell cytotoxicity mechanisms as well [52]. The final result of the cytotoxic activity is the target cell death, which can be achieved by necrosis and apoptosis [52]. Programmed cell death or apoptosis may be accomplished by nonsecretory mechanisms, through the interaction of effector and target cell membrane molecules, such as FasL-Fas, or by secretory mechanisms, mediated by degranulation and exocytosis of cytotoxic molecules from the cytotoxic cells’ cytoplasmatic granules, such as perforin, granzymes, and granulysin [52,53]. Previous studies have demonstrated increased granzyme B, perforin, and granulysin levels in lesional skin and peripheral blood of patients with psoriasis [54,55,56,57,58]. T lymphocytes’ subpopulations also have functional flexibility, converting Th17 and Treg to Th1 or Tc17 to Tc1 subpopulation [50]. Other subpopulations of Th lymphocytes, such as Th9 and follicular CD3+CD4+CXCR5+ T lymphocytes, also contribute to the pathogenesis of psoriasis, most likely by potentiation of existing immune events, especially the IL-17 pathway [34].

Regulatory CD4+CD25+ T-cells (Tregs), which maintain immune tolerance by secreting inhibitory cytokines and inducing apoptosis, are damaged in psoriasis and cannot suppress the proliferation of effector T lymphocytes [59]. The number of Tregs in the peripheral blood of patients is reduced compared to healthy controls [60].

Tissue-resident memory T-cells (Trms) remain in the skin long after the withdrawal of psoriatic changes, thus contributing to the appearance of lesions at the same sites during disease exacerbation [61]. Trms mediate local inflammation by secretion of IL-17 [61] and can be of the CD8+ phenotype in the psoriatic epidermis or the CD4+ phenotype when inhabiting the dermis [62].

Gamma delta (γδ) T lymphocytes have a γδ T-cell receptor and share the properties of adaptive and innate immunity [63]. Two γδ T-cells’ subpopulations, dermal γδ T lymphocytes and circulating Vγ9Vδ2 lymphocytes, have been found in psoriasis [64]. The number of dermal γδ T lymphocytes is increased in psoriatic skin [65]. These cells share many characteristics with Th17 and Tc17 lymphocytes, as they possess the IL-23 receptor, to whose stimulation they respond by IL-17 and IL-22 secretion and subsequent keratinocyte activation [63]. The discovery that γδ-positive T lymphocytes produce significantly higher amounts of IL-17 in the lesional dermis, compared to those that are γδ-negative, confirmed their importance in the psoriasis pathogenesis [65,66]. The number of Vγ9Vδ2 T lymphocytes in the patients’ blood is significantly reduced, indicating the possibility of their rapid recruitment to inflamed tissue, while their number normalizes after the use of adequate systemic therapy [67,68].

The remaining nonclassical lymphocytes, which contribute to the pathogenesis of the disease by releasing IL-17, TNF-α, IFN-γ, and other inflammatory cytokines and chemokines, are NK and NKT cells, ILC cells, and the so-called mucosal-associated invariant T (MAIT) lymphocytes [63,69].

#### 4.1.4. NK and NKT Cells

The essential functions of natural killer (NK) cells are the removal of infected and damaged cells in a cytotoxic manner [70] and the secretion of cytokines IFN-γ, TNF-α, and TGF-β upon their activation by IL-12 [44]. Recently, NK17 and NK22 cells, producing IL-17 and IL-22, have also been discovered [70]. In humans, there are circulating (cNK, CD56+CD16+) and resident NK tissue cells (rtNK, CD56+CD16−) [70], which are present in the psoriatic dermis as immature CD56bright cells, having less cytotoxic potential and more efficient cytokine secretion, in regard to CD56dim subtype [71]. It has been proven, in an in vitro experiment, that lesional NK cells with an expressed CD69 activation marker produce large amounts of IFN-γ and TNF-α, which then activate and stimulate keratinocytes on CXCL10 and CCL5 release. Due to the expression of their chemokine receptors on NK cells, the new NK cells have been attracted to the site of inflammation [72]. There is evidence that CD56dim cells are recruited in response to chemerin, whose expression is increased in psoriatic lesions [73]. In conducted investigations, a decreased number of NK cells, not correlated with the clinical severity of the disease, has been detected in the psoriasis patients’ circulation, while in newly diagnosed patients, their number was identical to that in healthy controls [74]. The study by Duphny et al. found that NK cells have impaired degranulation and cytokine production function [75]. The role of NK cells in the pathogenesis of psoriasis was further confirmed by the discovery of KIR-receptor gene polymorphisms (from killer-cell immunoglobulin-like receptors) [76]. Although it has been thought that NK cells mediate the immune response at the site of psoriatic inflammation by cellular cytotoxicity mechanisms and cytokine production, then participate in the adaptive immune response through DC modulation, and finally perform immune regulation by killing immature or overactive cells, such as macrophages and T lymphocytes, their functions need to be further investigated [75].

Natural killer T-cells (NKTs) are unique cells that, in addition to NK cell markers, also have T lymphocytes’ properties, more precisely their small diversity αβ-TCR [70]. Invariant NKT (iNKT) cells, which form the largest part of the total population [77], express receptors for IL-12, IL-18, IL-23, IL-25, and IL-33 [77]. Activation of NKT lymphocytes occurs by recognition of glycolipids by CD1d antigen-presenting molecules, similar to those of MHC group I, with consequent production of cytokines IFN-γ, TNFα, IL-10, IL-4, IL-13, IL-17, and GM-CSF [70]. Mature NKT1 cells release high levels of IFN-γ, NKT2 cells secrete IL-4, while so-called NKT17 cells produce IL-17A, IL-17F, and IL-22 [77]. NKT lymphocytes also express the chemokine receptors CXCR3, CCR5, and CCR6, which enhance their mobilization into the skin [34]. The NKT cells’ function can be altered by the TCR signal’s intensity modulation. Therefore, a single stimulus of the CD1d molecule results in increased production of IFN-γ, which contributes to cytotoxicity by CD8+ T lymphocyte production and NK cell activation, while repeated stimulations lead to increased IL-10, which mediates the regulatory function of NKT cells [78]. NKT cells probably do not form a central part of the psoriasis pathogenesis but are undoubtedly involved in it [77]. The results of an in vitro experiment in which cocultivation of NKT and CD1d + keratinocytes directly affected IFN-γ production [74] were confirmed by the in vivo discovery of significantly increased CD1d expression in patients’ lesional keratinocytes [79]. Studies with SCID mice have shown that psoriasis can be induced by injecting activated iNKT cells into a transplant of unaltered patient’s skin [80]. iNKT cells are present in psoriatic skin in increased numbers [81], while in the patients’ blood, their reduced or equal number was found compared to controls [74]. Increased blood levels of iNKT cells with pronounced inhibitory receptors correlated with disease severity [82]. Although the function of NKT cells in psoriasis is mainly unknown, it is thought that they contribute to disease progression by interacting with CD1d+ keratinocytes and producing IFN-γ and other cytokines, which mobilize T17 lymphocytes [77].

#### 4.1.5. Keratinocytes

Keratinocytes are the building blocks of the epidermis that, in addition to their structural and protective role, also possess immune functions [40]. Keratinocytes are equally important in mediating inflammation in the early and late stages of psoriasis, since they control the innate immune response, through the secretion of innate immune system molecules such as AMP, and the adaptive immune response, through the recruitment of T lymphocytes to the inflammation site [40]. As keratinocytes possess receptors for most pathogenic cytokines, the epidermis is the target tissue of psoriatic inflammation. Due to its increased proliferation and impaired differentiation, by the process of so-called regenerative maturation, the development of a characteristic psoriatic phenotype occurs [34]. Each immune cell’s cytokine leads to a specific keratinocyte response [34]. Since keratinocytes exhibit most receptors for IL-17, IL-22, TNF-α, IL-19, and IL-20, cytokine IL-17 exerts the most pronounced effect on the epidermis, causing epidermal hyperplasia and stimulating further keratinocytes’ proinflammatory molecules’ production, thus enhancing the inflammatory process in the skin [83]. Namely, keratinocytes respond to executive cytokines by dynamic production of several proinflammatory products, such as cytokines (TNF-α, IL-1β, IL-6, IL-17C, IL-19, IL-36γ), chemokines (CCL20, CXCL1, CXCL2, CXCL8–11), growth factors (EGF, VEGF), and AMP, by which they achieve positive feedback activation and mobilization of immune cells in the skin [34,84]. Although keratinocytes constitutively express some AMPs, epithelial injury releases keratinocytes’ effector innate immunity molecules such as the initiators of the psoriatic pathogenic process LL37, as well as β-defensins and S100 proteins, with chemotactic abilities [40]. In addition to having direct antimicrobial activity, AMPs increase the production of keratinocyte cytokines, IL-6 and IL-10, and chemokines, CXCL8 and CXCL10, which mobilize neutrophils, Th1 lymphocytes, and macrophages, and CCL20, which recruits mDCs and IL-17-producing cells at the site of inflammation, contributing to disease maintenance [85]. Increased AMPs levels in psoriasis successfully reduce after the use of systemic therapy [85]. Keratinocyte’s IL-1β affects the production of TNF-α, stimulates the activation of T lymphocytes, increases the expression of leukocyte selectins, and, together with IL-18, is involved in the differentiation of Th1 and Th17 lymphocytes [40]. VEGF secreted by keratinocytes in an inflammation state promotes angiogenesis with the consequent formation of vascular plaque, while its excessive expression in the mouse skin leads to the formation of psoriatic lesions [86]. The previously described model considers keratinocytes as the secondary participants of the psoriasis pathogenesis. In contrast, after the discovery of their gene alterations, they have been put in first place by the concept of aberrant keratinocyte biology [87]. It has been observed that epidermal expression of STAT3 in a transgenic mouse, activated by the IFN-γ, IL-6, IL-20, IL-17A, and IL-22 cytokines, causes psoriasis [83]. STAT3 is likely a key transcription factor and the link between the keratinocytes’ and immune cells’ interaction in the development of a psoriatic lesion [83].

### 4.2. Main Cytokines Involved in Psoriasis Inflammatory Networks

Numerous mediators, which are interconnected in different pathogenic circles, are involved in the initiation and maintenance of psoriasis [34]. Attitudes about the “main” cytokine of the psoriatic inflammatory process alternated with the advancement of knowledge and cognition. While Th1 lymphocytes were considered central cells of pathogenesis, IFN-γ, a cytokine of the IL-12/Th1 axis, which was found in high concentrations in the skin and blood of patients, was put in the foreground [88]. The role of IFN-γ in psoriasis has been demonstrated by the lesion formation after injection of this cytokine into the patient’s unaltered skin, transplanted to SCID mice, and after the discovery that it enhances the expression of about 400 genes in psoriatic skin by activating the STAT1 pathway [89]. It is produced by Th1 lymphocytes and NK cells [40]. Concurrently, it achieves its effect by activating DC and stimulating the release of adhesion molecules from keratinocytes, facilitating the mobilization of T lymphocytes into inflammatory plaques [40,90]. IFN-γ is an important cytokine of the early stage of psoriasis, while it has no major effect on the maintenance of the disease, since its direct blockade did not achieve the lesions’ withdrawal [91].

The observation that interferon therapy for hepatitis worsens psoriasis has confirmed the role of IFN-α in the pathogenesis of the disease [92]. This cytokine is considered a psoriasis initiator, as it mediates the maturation and activation of mDCs with the consequent release of IL-12, IL-15, IL-18, and IL-23 [27]. The production of large amounts of IFN-α is characteristic for acute forms of the disease, especially erythrodermic psoriasis [27]. Although IFN-α blockade in mouse models of disease prevented the development of psoriasis, the same effect was not achieved in clinical practice [39,93].

Recent knowledge holds that the basis of psoriasis immunopathogenesis is consisted in the IL-23/Th17 axis, where IL-23 affects the differentiation and activation of Th17 lymphocytes, which by secreting IL-17 exert their effects on keratinocytes and create a specific disease phenotype [61]. IL-23 is an IL-6/IL-12 cytokine family member, which consists of p19 and p40 subunits [94]. Although macrophages, keratinocytes, and LCs secrete it, its main sources are mDCs [94]. By binding to receptors (IL-23R), expressed on memory T lymphocytes, NK cells, neutrophils, mastocytes, macrophages, and ILC, IL-23 activates the STAT3 pathway in them [94]. IL-23 is a major factor for the survival of T17 lymphocytes and, as such, controls the expression of key cytokines for keratinocyte proliferation, i.e., IL-17A, IL-17F, IL-22, and IL-21. It increases the expression of TNF-α in macrophages, and IL-23R as well [95]. The role of IL-23 as a central cytokine in the pathogenesis of the disease was confirmed by functional studies of the development of psoriatic lesions after intradermal administration of IL-23 in mice [96], GWAS studies that revealed candidate genes involved in this pathway [97], increased IL-23 levels in the patients’ lesional skin and serum [98] and the clinical success of the biological drug ustekinumab [99].

The central role of IL-23 is directly related to IL-17A (IL-17), a member of the IL17A-F cytokine family, which due to its pronounced biological and inflammatory activity, has been recognized as a relevant factor in psoriasis immunopathogenesis [100]. Although its primary source is Th17 lymphocytes, other cells, such as Tc17, γδ T lymphocytes, NK cells, macrophages, mastocytes, neutrophils, and ILC, also contribute to its production [83]. After binding of IL-17 to IL-17R, the transcription via CCAAT/enhancer-binding protein is activated, acting on endothelial cells, fibroblasts, and especially keratinocytes, stimulating their proliferation and production of AMPs and proinflammatory cytokines (IL-1, IL-6, IL-19, IL-23, IL-36γ) [83]. Additionally, by inducing IL-8, it maintains neutrophil mobilization and activation and acts as a chemoattractant for DC, T lymphocytes, and NK cells [101]. The role of IL-17 was supported by functional studies in which psoriatic lesions followed its intradermal administration in mice [102] by discoveries of its elevated levels in patient’s lesional and nonlesional skin and serum [103] and its induction of 600 genes’ expression [104]. The studies likewise found that the effects of UVB phototherapy and anti-TNF-α drugs are achieved by suppressing the IL-17 signaling pathway and by the excellent clinical efficacy of anti-IL-17A drugs [105,106]. IL-17, in synergistic cooperation with IL-22 and TNF-α, has been shown to stimulate the production of inflammatory cytokines and AMP in keratinocytes [101].

IL-22, a member of the IL-20 family, is produced, under the influence of IL-23, by Th22 and Th17 lymphocytes and primarily acts on keratinocytes [37]. In psoriasis, in addition to proinflammatory synergism with IL-17 and TNF-α, it interacts with IFN-α, which enhances the expression of its receptor (IL-22R) on keratinocytes [107]. The role of this cytokine in psoriasis has been confirmed by its increased expression in lesional skin and circulation, which correlates with disease severity [40,108]. Although the mouse model showed that epithelial hyperplasia caused by IL-23 is also dependent on IL-22, and subsequently IL-22/Th22 axis theses have been developed [109], still its therapeutic blockade has not been successful [110], thus IL-22 is probably not a crucial part of the psoriasis pathogenesis.

The innate immunity cytokine, TNF-α, is elevated in patients’ serum and psoriatic skin [104]. It is produced by keratinocytes, macrophages, DCs, and T lymphocytes, and its receptors, which are present in practically all body cells, activate NF-κB, MAPK (from mitogen-activated protein kinase), and JNK (from c-Jun N-terminal kinase) pathways [111]. The key effect of TNF-α is to stimulate the production of IL-23 by DCs, for what is considered a cytokine superior to the IL-23/IL-17 axis [112]. Anti-TNF-α drugs, known as pioneers of the biological therapy of psoriasis, mediate their action by impairing the interaction of DCs and T lymphocytes, i.e., by preventing the synthesis of IL-23; therefore, their clinical effect is mostly associated with suppressing the IL-23/Th17 axis [112]. The cognition that the mentioned innate and adaptive immune cells and their cytokine and chemokine network form the “skin immune system” participating in psoriatic pathogenetic events, contributed to the development of the new, promising, multidisciplinary science called nanodermatology, offering the personalized approach of treating psoriasis [113].

## 5. Conclusions

Psoriasis is a recurrent, chronic, T-cell-mediated, polygenic disease characterized by the appearance of erythematosquamous plaques in certain predilection sites such as the scalp, extensor parts of the extremities, especially the elbows and knees, and the lumbosacral area. Psoriasis is a relatively common disease that significantly impairs patients’ quality of life, carries the risk of many comorbidities, and, therefore, oftentimes shortens life expectancy. In addition, psoriasis has a noticeable socioeconomic impact on society in general. Currently, it is more often referred to as a psoriatic disease, since newer studies have shown that inflammation is present beyond the skin and that upgraded levels of pathogenic, psoriasis-specific mediators are detectable in patients’ circulation as well. Intensive research conducted in the field of psoriasis for more than six decades revealed that the underlying pathogenetic mechanisms are marked by intense and intertwined inflammatory events, mediated mainly by T lymphocytes, dendritic cells, and keratinocytes, contained in the so-called IL-23/Th17 axis. Therefore, psoriasis is today considered a prototype of Th17 disease. Advances in psoriasis immunopathogenesis knowledge have led to the successful development of new targeted, biological drugs that caused significant improvements in the clinical picture and patients’ quality of life. Even though many parts of the complex psoriasis pathogenesis have been revealed, new future research will undoubtedly be needed to supplement these findings. In times to come, the aspiration for personalized medicine development, where drugs will be used tailored to each patient, their own genetic mutations, immune system dysfunctions, and clinical manifestations of the disease, will be expressed. Therefore, therapeutic approaches are moving towards precision medicine that is more respectful to the patient’s biological fingerprint. Accordingly, even more extensive knowledge of immunopathogenesis will be necessary in order to be able to act on multiple key target sites and to achieve maximum results in the treatment of psoriasis and diseases with similar pathogenetic mechanisms.

## Data Availability

Not applicable.
